# Healing of the Acutely Injured Anterior Cruciate Ligament: Functional Treatment with the ACL-Jack, a Dynamic Posterior Drawer Brace

**DOI:** 10.1155/2016/1609067

**Published:** 2016-12-07

**Authors:** Matthias Jacobi, Nikolaus Reischl, Karolin Rönn, Robert A. Magnusson, Emanuel Gautier, Roland P. Jakob

**Affiliations:** ^1^Orthopädie Rosenberg, Rosenbergstrasse 150, St. Gallen, Switzerland; ^2^Department of Orthopedic Surgery, HFR Hôpital Cantonal, Fribourg, Switzerland; ^3^Private Clinic Hansa Graz, Körblergasse 42, 8010 Graz, Austria; ^4^Schulthess Klinik, Lengghalde 2, Zürich, Switzerland; ^5^Department of Orthopaedic Surgery, Sports Health and Performance Institute, The Ohio State University, Columbus, OH, USA; ^6^En Chambaz, Môtier, Switzerland

## Abstract

*Background*. The injured anterior cruciate ligament (ACL) has a limited healing capacity leading to persisting instability.* Hypothesis/Purpose*. To study if the application of a brace, producing a dynamic posterior drawer force, after acute ACL injury reduces initial instability.* Study Design*. Cohort study.* Methods*. Patients treated with the ACL-Jack brace were compared to controls treated with primary ACL reconstruction und controls treated nonsurgically with functional rehabilitation. Measurements included anterior laxity (Rolimeter), clinical scores (Lysholm, Tegner, and IKDC), and MRI evaluation. Patients were followed up to 24 months.* Results*. Patients treated with the ACL-Jack brace showed a significant improvement of anterior knee laxity comparable to patients treated with ACL reconstruction, whereas laxity persisted after nonsurgical functional rehabilitation. The failure risk (secondary reconstruction necessary) of the ACL-Jack group was however 21% (18 of 86) within 24 months. Clinical scores were similar in all treatment groups.* Conclusion*. Treatment of acute ACL tears with the ACL-Jack brace leads to improved anterior knee laxity compared to nonsurgical treatment with functional rehabilitation.

## 1. Introduction

The acutely injured anterior cruciate ligament (ACL) has a poor healing capacity, resulting regularly in persistent instability of the knee [1, 2]. Surgical reconstruction has become an accepted treatment to restore ACL stability in the younger and more active patient [3]. The reason that ACL healing is uncommon is not fully understood, but biological, biomechanical, and anatomical factors all likely contribute [4, 5]. The ACL, in contrast to extra-articular ligaments, does not form a fibrin-platelet clot to initiate tissue healing. Clot formation is likely inhibited by factors in the surrounding synovial fluid [4, 6]. Further, during rehabilitation and normal daily activities following ACL injury, the quadriceps-induced anterior drawer and other movements of the knee can pull the ligament stumps apart [7], potentially resulting in a lengthened ligament even in cases in which healing does occur. Finally, the positions of the ligament stumps may be such that there is no contact between them after injury, effectively preventing healing.

The ACL does have characteristics that could promote healing. For example, the ligament is well vascularized, which is required for tissues healing [8, 9]. Different methods have been undertaken to enhance healing of the ACL, including primary suture repair, healing response techniques, immobilization, bracing, and supplementation with scaffolds, growth factors, and collagen-platelet composites [5, 10–14]. Although primary ACL suture has been shown to improve laxity in the short term, it has shown a high failure rate with longer follow-up [5, 11, 14]. Several functional knee braces have been evaluated and noted not to affect knee anterior laxity [10, 15, 16]. Fujimoto et al., however, showed in a group of 31 patients with low athletic demands that bracing with an extension block improved stability in 74% of patients, but 26% of the patients went on to require ACL reconstruction [17]. Biologic strategies to enhance ACL healing are quite promising, but only in vitro and animal studies are available currently [4, 5]. Internal stabilization techniques report promising results. They also rely on the self-healing of the injured ACL [18–22].

The purpose of the present study was to assess whether ACL healing and final knee laxity can be improved in patients with acute ACL injuries by altering the biomechanical conditions during healing through the use of a brace producing a dynamic posterior drawer force. We hypothesized that (1) ACL healing and anterior laxity of the knee are improved through the use of the ACL-Jack brace relative to a control group with no brace and (2) that in patients in whom use of the ACL-Jack brace results in satisfactory knee function anterior laxity is equal to that achieved with primary ACL reconstruction.

## 2. Patients and Method

### 2.1. Inclusion

From March 2004 to February, 2009, 86 patients with acute ACL injury were enrolled in a prospective study at our institution to evaluate the effectiveness the ACL-Jack brace for management of acute ACL injuries. Additionally 40 patients were enrolled to compare the treatment with the ACL-Jack brace to two standard treatments (20 patients each). Patients were recruited and enrolled with the following inclusion criteria:

- acute injury (<3 weeks),
- complete or subtotal ACL tear confirmed clinically and with MRI,
- informed consent of the patient about the planned therapy including possible complications and drawbacks,
- patients with associated grade I or II MCL injury included.

Additional treatment was provided in the following situations:

- In case of meniscal tears, either a partial meniscectomy or meniscal repair was performed prior to bracing.
- If the ruptured ACL showed anteriorly displaced fibers on MRI, these fibers were reduced arthroscopically.

Exclusion criteria were

- patients unwilling to follow the treatment protocol or inability to comprehend it,
- ACL injuries older than 3 weeks,
- associated injury of the PCL, LCL, MCL (grade III), or any other lesion requiring surgery.

Allocation to the groups relied on patient's choice after informed consent.

### 2.2. Study Groups

#### 2.2.1. ACL-Jack Group

Patients in this main study group were treated with the ACL-Jack brace. The prefabricated brace was adjusted by an orthopaedic technician and worn for three months day and night and for an additional month during daytime only. Full weight bearing was allowed from the start of the treatment. Range of movement to the extent possible in the brace was allowed, giving patients a range of flexion of about 0° to 100°. Removal of the brace was allowed in 90° of knee flexion (sitting position) without quadriceps contraction. With the knee in flexion it was also the recommended position to take a shower. Special attention was given to the instruction to patients with written information and regular assessment of compliance in the initial phase of treatment. After four months, the brace was removed and exercises and physiotherapy were started to aid the recovery of muscle strength and full mobility. Sporting activity, including cutting and pivoting, was allowed after six months. Patients received thromboprophylaxis during the first four weeks of treatment with low-molecular-weight heparin due to the compressive nature of the brace.

#### 2.2.2. Functional Treatment Group

This group underwent a functional rehabilitation protocol in physiotherapy (muscle strengthening coordination and proprioception program) without any brace for 2 to 4 months.

#### 2.2.3. Primary ACL Reconstruction Group

This group underwent primary reconstruction with an anatomic single bundle (patellar tendon) technique. Tunnels were drilled on the femoral side with an outside-in drill guide. Femoral fixation was performed with a press fit technique (conical bone block in a conical tunnel). Tibial fixation was accomplished with an interference screw.

For subgroup analysis groups were divided in successfully treated and failed patients if necessary.

### 2.3. Characteristics of the Brace

**Figure 1:**
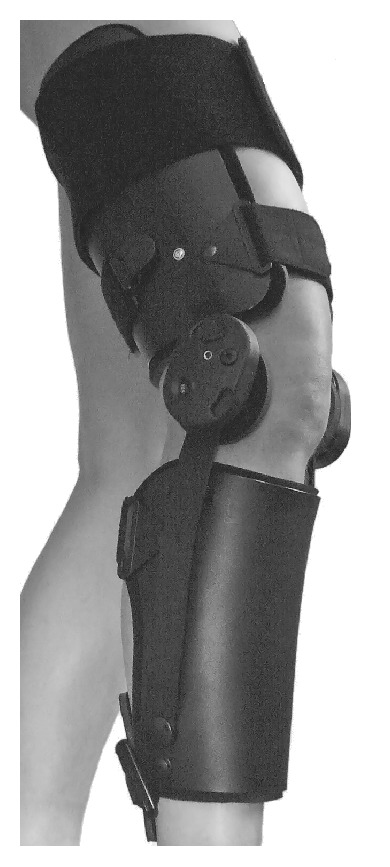
Photograph of the ACL-Jack brace.

**Figure 2:**
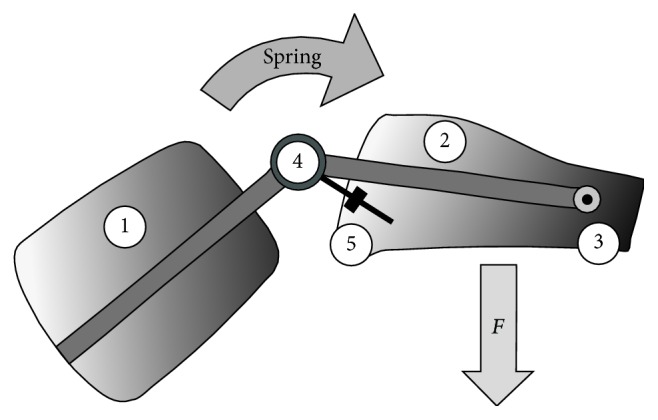
Diagram showing that the brace consists of an upper thigh (1) and a leg part (2) connected through a hinge at the ankle (3) and knee (4). The load is applied through a relocatable load arm (5) from the hinge to the leg part, which rotates around the distal hinge (3). *F* = force.

The ACL-Jack is a brace producing a dynamic posterior drawer with built-in springs that apply a posteriorly directed force to the anterior proximal tibia (Figures 1 and 2) inversely to the PCL-Jack brace [23]. This force opposes the quadriceps-induced anterior drawer that can occur in the ACL deficient knee. The brace consists of thigh and leg sections that are connected through a hinge at the knee and ankle. The hinge at the knee allows flexion and extension movement and the hinge at the ankle allows independent posterior translation. The force is applied from the hinge at the knee through a load arm to the leg splint. The particular feature of the brace is that knee movement is disengaged from force transmission. The spring inside the hinge at the knee can be loaded up to 15 positions, each unit increasing the translation force. In general, 12 (first 2 weeks 10 units) units were chosen, corresponding to a posterior force of 6 kg to 7 kg. The force is maintained throughout full range of movement [24]. To reduce the direct pressure to the anterior rim of the tibia the prefabricated brace was adapted in a way that the main pressure was applied medial and lateral to the tibial metaphysis. Additionally the anterior skin and soft tissues were protected by pads.

### 2.4. Initial Assessment

All patients underwent routine clinical examination of the knee. Anterior knee laxity was assessed using the Rolimeter arthrometer (Aircast; DJO, Vista, California) in 20° of knee flexion in comparison with the opposite side. Every knee was evaluated with MRI with a special focus on anteriorly displaced fibers of the injured ACL. Examinations were done by the first or the senior author. Preinjury patient-reported knee scoring was done using the Lysholm knee function scoring scale [25], the Tegner activity level rating scale [26], and the International Knee Documentation Committee (IKDC) knee scoring system [27].

### 2.5. Follow-Up Assessment

For all studied groups, scheduled follow-up appointments took place at six, 12, and 24 months. They involved clinical examination of the knee, bilateral comparative Rolimeter arthrometry. Examinations were done by the first or the senior author. An MRI was performed after six months and evaluated by an independent radiologist. MRI was not performed in the primary ACL reconstruction group. Patients completed the follow-up by evaluation using the Lysholm scale, the Tegner scale, and the IKDC Score at 12 and 24 months.

### 2.6. Statistical Analysis

Data are presented as mean, standard deviation, and range. Due to data distribution, nonparametric analysis techniques were utilized, including the Wilcoxon signed-rank test, Kruskal-Wallis test, and Friedman test. A *p* value ≤ 0.05 was considered to be significant. Calculations and graphs were performed using MedCalc Software version 10.4.8.0 (MedCalc Software Buba, Mariakerke, Belgium).

## 3. Results

### 3.1. Baseline Data

The ACL-Jack group consisted of 86 patients, of which 84 (98%) had complete follow-up. One patient moved abroad and one other was lost to follow-up. Of the 84 remaining patients 18 (21%) required a secondary ACL reconstruction due to persistent and disabling instability (*n* = 13) or repeat injury (*n* = 5) within 24 months (Table 1).

The functional treatment and the primary ACL reconstruction group consisted both of 20 patients and had 100% follow-up. Six patients (30%) in the functional treatment group required secondary ACL reconstruction due to disabling instability within 24 months. No recurrent instability episodes or revision ACL reconstructions occurred in the primary ACL reconstruction group (Table 1).

As allocation to the groups was based on patients choice, patients treated with the ACL-Jack and the functional treatment group were both significantly older (*p* = 0.00002) and had a higher female to male ratio than the primary reconstruction group (Table 1).

The highest failure risk was observed among young men with higher level sport activities on the Tegner scale (Table 2).

### 3.2. Comparative Side-to-Side Anteroposterior Stability

Anteroposterior stability was evaluated with the Rolimeter in Lachman position. At the initial assessment patients showed a mean side-to-side difference of 4 to 5 mm in all groups. There was no statistical difference between the groups (*p* = 0.32). At 24-month follow-up a significant improvement of anterior knee laxity was observed in patients treated successfully in the ACL-Jack group and in the primary ACL reconstruction group (*p* = 0.000002) with a residual laxity of average 1 mm. In the ACL-Jack group 55 (83%) had a residual laxity ≤2 mm, nine (14%) 3-4 mm, and two (3%) ≥5 mm. In the functional treatment group the initial degree of laxity persisted (Table 3).

### 3.3. Clinical Scores

Clinical outcome was evaluated with the Lysholm scale and IKDC Score and the activity level with the Tegner scale. All study groups showed a significant decrease of the IKDC Score and Lysholm scale of about 10%. A comparison between the groups showed no significant differences at all-time points (Table 3). A correlation between score outcome and laxity was not found. Activity level decreased 0.7 points on the Tegner scale for patients treated successfully in the ACL-Jack group and the primary ACL reconstruction group and 1.6 points for the functional treatment group. 39 of the 66 patients in the ACL-Jack group (59%) reached on the Tegner scale the preinjury activity level.

### 3.4. MRI Findings

**Figure 3:**
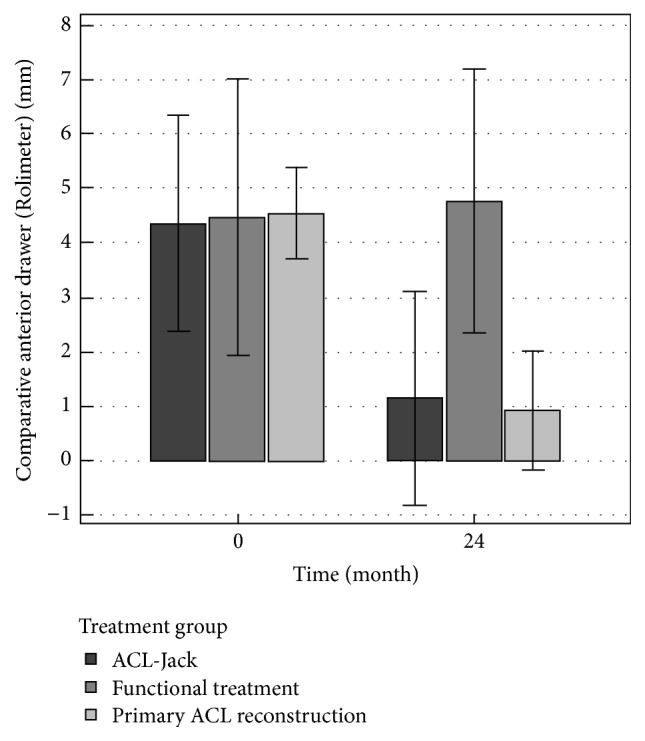
Bars comparing the initial (0) and follow-up (24-month) anterior drawer (bilateral comparison) measured with the Rolimeter of the three treatment groups. Significance is reported in Table 3.

**Figure 4:**
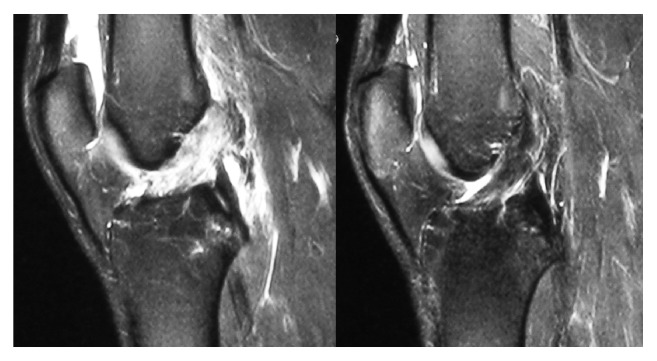
Initial and follow-up MRI six months after treatment with the ACL-Jack brace.

Healing of the ligament was documented on MRI after 6 months (Figure 3). We rated continuity and thickness of the ACL. In knees treated successfully with the ACL-Jack brace (*n* = 66) a normal appearing ACL was found in 36 (55%) knees (Figure 4). Twenty-five knees (38%) had preserved continuity of the ACL but either an irregular appearance or a thinner remnant or both. Another five (7%) showed only minimal remnants of the ACL. None of the failed patients in the ACL-Jack group had a normal appearing ACL. Two patients (14%) treated successfully in the functional treatment group had a normal appearing ACL. Four (28%) had preserved continuity of the ACL but either an irregular appearance or a thinner remnant or both. Another eight (57%) showed only minimal remnants of the ACL. None of the failed patients in the functional treatment group had a normal appearing ACL.

### 3.5. Complications

Within the ACL-Jack group the main complication encountered was skin problems at the anterior tibial rim due to the posteriorly directed force of the pad (*n* = 10). Furthermore one case of arthrofibrosis occurred. In the primary reconstruction group one case of arthrofibrosis was treated arthroscopically and in one patient an interference screw had to be shortened.

## 4. Discussion

The main finding of this study is that patients with acute ACL injury treated with the ACL-Jack brace show improved healing on MRI and an improved AP knee laxity compared to an unbraced control group. Final laxity was in the successfully treated ACL-Jack group comparable to the primary reconstruction group. However 21% of the patients in the ACL-Jack group underwent secondary ACL reconstruction within 24 months due to persistent instability or repeat trauma. Patients who failed in the ACL-Jack group were significantly younger, more active, and mostly men. In the functional treatment group the failure risk was even higher (30%) although the average activity level on the Tegner scale was lower. Hypothetic reasons leading to failures in the ACL-Jack group are lack of compliance, insufficient reposition of the ACL fibers, biological factors, and mechanical reasons. Fujimoto et al. showed that bracing with an extension block improves stability [17]. Therefore it can be hypothesized that addition of an extension block to the ACL-Jack brace would alter the results.

Clinical outcomes measured with the IKDC Score and Lysholm scale were similar for all treatment groups and did not correlate with anterior knee laxity. However the scores were calculated without the failed patients in the ACL-Jack and functional treatment group, which would have worsened the results in these groups if these failures were considered.

This study has several limitations. First and most important, allocation to the different study groups was based on each patient's choice after informed consent. Therefore, there was a tendency that younger and more athletic patients were included in the primary reconstruction group whereas older and less athletic patients regularly chose the ACL-Jack or the functional treatment group. These differences in groups limit conclusive comparison of outcomes. We however assume that comparison of knee laxity is meaningful within the three groups. Furthermore no information is available about biomechanical strength of the healed ACL. At least five patients sustained a new relevant injury with recurrent instability. It is unknown whether the healed ligament has lower strength and is more prone to such reinjury. Finally, while efforts were made to ensure patient compliance with the ACL-Jack brace, compliance was not directly monitored and is not known.

## 5. Conclusion

The results of this study show that healing of the freshly injured ACL may be better than generally assumed, particularly when the biomechanical environment is enhanced with specific bracing methods. While a significant proportion of patients still required ACL reconstruction when braced, ACL-Jack brace use significantly improved anterior knee laxity relative to an unbraced control group. The use of bracing to enhance the biomechanical healing environment for ACL healing may play a role as primary ACL healing gains new consideration due to advances in biologic mediators of healing such as platelet-rich plasma, stem cells, tissue augments, and internal stabilization techniques.

## Figures and Tables

**Table 1:** Baseline data of the ACL-Jack group, the functional treatment group, and the primary ACL reconstruction group.

		ACL-Jack	Functional treatment	Primary ACL reconstruction	*p*
Included patients					

Patients	*n*	86	20	20	
Dropouts (total)	*n* (%)	20 (23%)	6 (30%)	0	
(i) Failures	*n* (%)	18 (20.9%)	6 (30%)	0	
(ii) Lost to follow up	*n* (%)	2 (2%)	0	0	
Age	years	32 ± 14 (14–74)	35 ± 10 (21–48)	23 ± 7 (15–40)	0.00002
Sex	M/F	52/33	13/7	14/6	
Side	R/L	48/37	8/12	12/8	
Meniscus tear	*n* (%)	11 (12%)	0	6 (30%)	
ACL displaced	*n* (%)	28 (33%)	6 (30%)	—	
Injury to treatment	days	14 ± 10 (10–21)	—	37 ± 26 (10–89)	0.0001

Analyzed patients					

Patients	*n*	66	14	20	
Sex	M/F	36/30	8/6	14/6	
Side	R/L	38/28	5/9	12/8	
Meniscus tear	*n* (%)	9 (14%)	0 (0%)	6 (30%)	
ACL displaced	*n* (%)	23 (27%)	4 (29%)	—	
Injury to treatment	days	14 ± 8 (10–21)	—	37 ± 26 (10–89)	0.0001

**Table 2:** Comparative data of successfully treated and failed patients within the ACL-Jack group.

ACL-Jack group				
		Successful	Failures	*p*
Patients	*n*	66	18	
Age	Years	34 ± 15 (14–74)	24 ± 12 (15–57)	0.00002
Sex	M/F	36/31	16/2	
Meniscus tear	*n* (%)	9 (14%)	2 (11%)	n.s.
ACL displaced	*n* (%)	23 (27%)	5 (28%)	n.s.
Injury to treatment	Days	13 ± 5 (3–21)	14 ± 7 (5–21)	n.s.

**Table 3:** Clinical outcome and side-to-side ACL stability (Rolimeter) of the ACL-Jack (successful), functional treatment (successful), and primary ACL reconstruction group.

	ACL-Jack	*p*	Functional treatment	*p*	Primary ACL reconstruction	*p*	*p* intergroup
Tegner preinjury	6.6 ± 2 (4–10)	<0.00001	5.1 ± 1.4 (2–6)	<0.00001	8.6 ± 1.3 (5–10)	0.00026	0.00002
Tegner 12 months	5.6 ± 2.1 (3–10)		3.4 ± 0.9 (2–5)		7.7 ± 1.8 (4–10)		<0.000001
Tegner 24 months	5.9 ± 2 (3–10)		3.5 ± 1 (2–5)		7.9 ± 1.7 (4–10)		<0.000001

Lysholm preinjury	99.7 ± 1.2 (95–100)	<0.00001	100 ± 0 (100-100)	<0.00001	98.6 ± 2.4 (94–100)	<0.00001	0.047
Lysholm 12 months	92.8 ± 8.6 (67–100)		93.7 ± 6.3 (79–100)		88.4 ± 6.9 (79–100)		0.055
Lysholm 24 months	93.3 ± 8.3 (67–100)		92.7 ± 7.4 (67–100)		89.1 ± 7.7 (74–100)		0.034

IKDC preinjury	96.5 ± 5.2 (72–100)	<0.00001	97 ± 3.5 (91–100)	<0.00001	98.4 ± 3 (90–100)	<0.00001	0.17
IKDC 12 months	88.7 ± 9.4 (58–100)		85.2 ± 9.1 (66–100)		88.1 ± 8.4 (72–100)		0.72
IKDC 24 months	90 ± 8.7 (69–100)		86.4 ± 11 (66–100)		88.3 ± 8.6 (74–100)		0.37

Diff injury (mm)	4.3 ± 2 (3–11)	<0.00001	4.5 ± 2.5 (2–10)	0.71	4.6 ± 0.8 (4–6)	<0.0001	0.32
Diff 6 months (mm)	0.9 ± 1.8 (0–4.5)		—		—		—
Diff 12 months (mm)	1 ± 1.4 (0–4)		—		—		—
Diff 24 months (mm)	1.1 ± 2 (0–5)		4.8 ± 2.4 (2–8)		0.9 ± 1.1 (0–3)		0.000002
